# Assessment of Skeletal and Non-skeletal Fluorosis in Endemic Fluoridated Areas of Vidharbha Region, India: A Survey

**DOI:** 10.4103/0970-0218.66868

**Published:** 2010-04

**Authors:** Sudhir Rawlani, Shobha Rawlani, Shivlal Rawlani

**Affiliations:** Rajnandgeon, Chattisgarh, Sharad Pawar Dental College, DMIMS, Sawangi (M), Wardha, Maharashtra, India; 1 Department of Anatomy, Mahatma Gandhi Institute Medical Sciences Sewagram, Wardha, India; 2 Department of Oral Medicine and Radiology, Sharad Pawar Dental College, DMIMS, Sawangi (M), Wardha, Maharashtra, India

**Keywords:** Biochemical, hematology, morphological, radiology, skeletal fluorosis

## Abstract

**Objectives:**

To evaluate skeletal and non-skeletal fluorosis in patients living at endemic fluoridated areas and also the morphological changes in red blood cells (R.B.C.’s).

**Materials and Methods:**

The cross-sectional study was conducted at Vidharbha region of Maharashtra, India. An ethical clearance was obtained from the concerned authorities. Fifty families were screened and 204 subjects who had dental/skeletal fluorosis were included in the study. The aims and objectives were explained to the study subjects of the village and biochemical, hematological and radiological assessment was done. The main source of drinking water in this area was tube well. The concentrations of fluoride in two different areas of same village were 4 and 4.5 ppm.

**Results:**

Prevalence of skeletal fluorosis and non-skeletal fluorosis in male patients was 56.87% (116) and in female patients (88) it was 43.13%. RBC count in male patients was 5.03 ± 0.49 while in female patients it was 4.70 ± 0.47. With significant difference between male and female patients, *P* value was 0.003. Hb% in male patients was 12.44 ± 1.76 and in female patients it was 11.31± 1.34, showing significant difference between male and female patients *P* value 0.038. Alkaline phosphate level in male patients was 289.68 ± 149.09 and in female patients it was 276.68 ± 164.97. ESR count in male patients was found 11.41 ± 8.75 and in female patients it was 13.29 ±7.37. Radiological finding of fluorosis patients shows thickening of inner and outer tables of skull bone in 83.92% of patients and only 7.84% of the patients were suffering from barrowing of long bone.

## Introduction

In human nutrition, fluorine plays a dual role; to prevent dental caries at a certain level of intake and can cause serious damages in bony and dental tissues.(1) Skeletal changes and mottled enamel may result when drinking water content of fluoride exceeds 2 ppm.(2) Clinical studies have shown that fluoride intake rapidly enters mineralized tissues like bone and developing teeth.(3)

The estimated range of safe and adequate intake of fluorides for adults is 1.5 to 4.0 mg per day and it is less for children and those with renal disease.(4) The daily intake of fluoride in endemic regions varies from 10 to 35 mg and can be even higher in summer months. The adverse effects of fluoride include dental fluorosis, skeletal fluorosis and it also affects R.B.C. cell wall.

In skeletal fluorosis, the patients often complain of a vague discomfort and paresthesiae in the limbs and the trunk, pain and stiffness in the back appear next, especially in the lumbar region, followed by dorsal and cervical spines.(5) Restriction of the spine movements is the earliest clinical sign of skeletal fluorosis. The stage at which skeletal fluorosis becomes crippling usually occurs between 30 and 50 years of age in the endemic regions.(4)

Red blood cell membrane is an important structural entity, which lodges the chemical, factors responsible for blood group substances, considerable enquiry into the membrane structure. It is now known that when fluoride is ingested, it will also accumulate on the erythrocyte membrane, besides other cells, tissues and organs. The erythrocyte membrane in turn looses calcium content.(6) The membrane, which is deficient in calcium content, is pliable and is thrown into folds. The RBCs attain the shape of an ameba with pseudopodia-like folds projecting in different directions, which are termed as echinocytes. The echinocytes will be found in circulation in large numbers, depending upon the extent of fluoride poisoning and duration of exposure to fluoride.

Hence, the objectives of present study are to assess the prevalence of skeletal fluorosis, to evaluate the hematological, biochemical and radiological changes in skeletal and non-skeletal fluorosis in endemic fluoridated areas and to evaluate the morphology changes in red blood cells wall.

## Materials and Methods

The cross-sectional study was conducted at Pimpalgeon village, near Yavatmal Vidarbha region of Maharashtra. The main source of drinking water in this area is tubewell. The concentration of fluoride in drinking water was measured using ion selective electrode method using an orion-ptt meter. The concentration of fluoride in drinking water sources of two different areas of same village was found to be 4 and 4.5 ppm.

The study was conducted over a period of three months. Fifty families out of 2500 total population living in the village were screened for dental/skeletal fluorosis. Before the start of the study, an ethical clearance was obtained from the concerned authorities. The aims and objectives were explained to the study subjects. A written consent was obtained from all the study subjects. After screening the 50 families, 204 subjects were found to be affected with dental and skeletal fluorosis. All these study subjects were referred to Jawaharlal Nehru Medical College and Hospital for biochemical, hematological and radiological investigations.

The blood samples were collected from 204 subjects to investigate the hematological and biochemical changes that included the RBC count, hemoglobin concentration (Hb%) and erythrocyte sedimentation rate (ESR) and morphological evaluation of RBC. Biochemical investigations included were measurement of serum alkaline phosphates levels.

Each patient was subjected to radiographic examination of cervical spine and long bone in the Department of Radio-diagnosis, Jawaharlal Nehru Medical College and Hospital, Sawangi (M), Wardha for evaluation of skeletal flurosis.

Data was analyzed by using the statistical software SPSS (Windows versions 17). The independent-samples t-test was used to test the difference between genders.

## Results

In the present study, a total of 204 subjects were examined including 116 males and 88 females, the mean age of male subject was 17.4 years and that of female subject was 15.9 years. All the results obtained after various investigations were recorded and data was analyzed by using the statistical software SPSS, (Windows versions 17). The independent-samples t-test was used to test the difference between genders.

## Discussion

Endemic fluorosis has been reported from different parts of the country.(7,8) The etiological factor in the earlier report was the contamination of surface water by underground water because of change in soil strata where topsoil became underground soil and vice versa due to construction of a dam in a nearby area.(9,10) However, in the present study, fluorosis occurred due to consumption of water from deep bore wells. The deep bore wells were constructed in the village in the year of 1987 to 1995 with an average depth of 250 and 265 feet.

In the present study, Hb% in male patients was 12.44 ± 1.76 and in female patients it was 11.31 ± 1.34, which shows significant difference *P* value 0.037. These finding are similar to the findings obtained by Gupta S, Goel D, Singhal A in 2005.(11)

**Figure 1:**
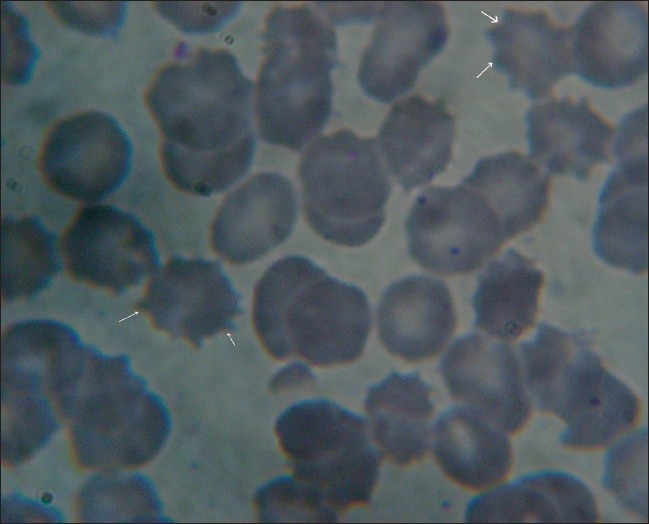
Showing RBC cell wall changes (Echinocytes)

The RBC membranes, which are deficient in calcium content, are pliable and are thrown into folds and attend the shape of an ameba called echinocytes.(12) Echinocytes undergo phagocytosis and are eliminated from circulation.(13) In the present study, echinocytes were found in 3.92% of patients may be because of chronic ingestion of fluoride i.e. for 16-year mean age of patients in our study [Figure 1].

While RBC count in male patients was 5.03 ± 0.49 and in female patients it was 4.70 ± 0.47, there is a significant difference between male and female patients’ *P* value 0.003. These findings correlate well with the findings obtained by Gupta S, Goel D, Singhal A in 2005.(11)

In the present study, ESR was raised in female patients (13.29±7.37) than male patients (11.41±8.75), which correlate well with the finding obtained by Gupta S. Goel D Singhal A, in 2005.(11) Alkaline phosphate level of the fluorite patients was revised in the present study. Alkaline phosphate level in male patients was 289.68 ± 149.09 and in female patients it was 276.68 ± 164.97, which is consistent with the findings of D. Reddy Raja, Rosenguist 1974, S.K. Gupta. R.C. Gupta 1993 also obtained similar results while study conducted by T. chakma S.B. Singh reported that alkaline phosphate level in fluorotic patients was within normal limits.(14,15)

**Figure 2:**
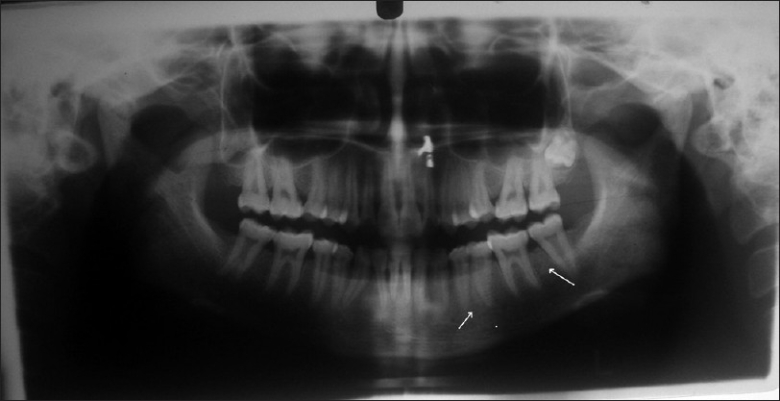
Jaw bone shows prominent marrow spaces

**Figure 3:**
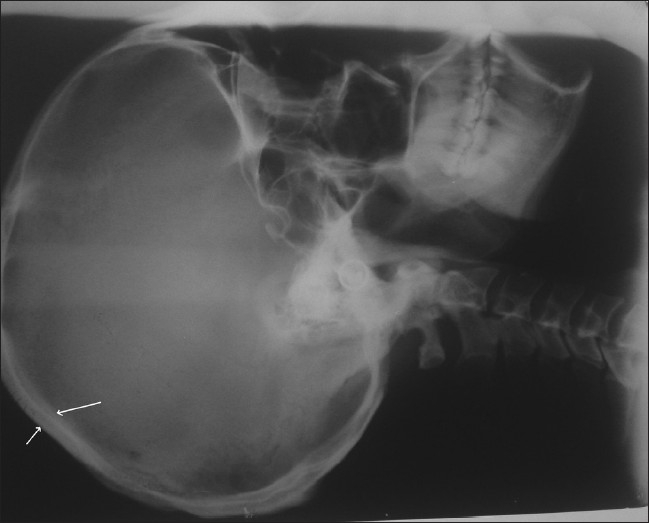
Showing barrowing of long bone

**Figure 4:**
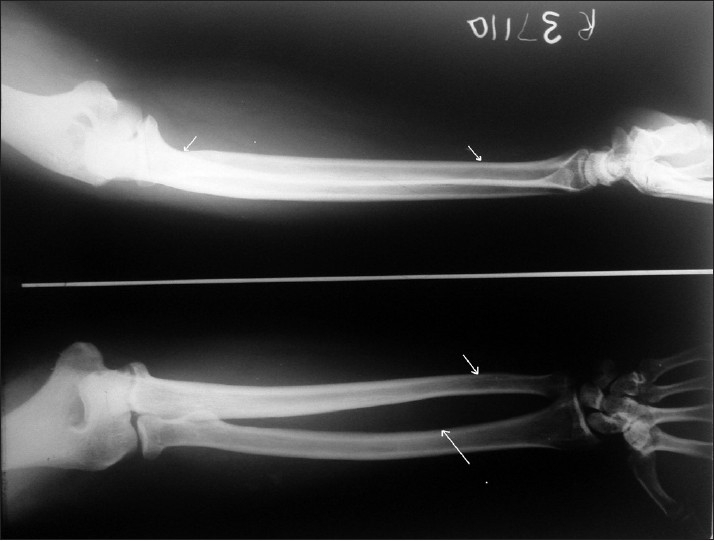
Skull bone showing thicken inner and outer table

Radiologic changes in jaw bone were seen in the form of prominent marrow spaces [Figure 2] In the present study, 7.84% had barrowing of long bone [Figure 3], while 83.92% shows thickened inner and outer table in skull radiograph [Figure 4]. These findings correlate with most of the studies conducted by Dr. Raja Reddy, T. Chakma. S.B. Singh and Vandana K.L. Sesha Reddy 2006.(4,15,16)
